# Avian intestinal ultrastructure changes provide insight into the pathogenesis of enteric diseases and probiotic mode of action

**DOI:** 10.1038/s41598-020-80714-2

**Published:** 2021-01-08

**Authors:** Shaniko Shini, R. Claire Aland, Wayne L. Bryden

**Affiliations:** 1 School of Agriculture and Food Sciences, University of Queensland, Gatton, QLD 4343 Australia; 2 School of Biomedical Sciences, University of Queensland, St Lucia, QLD 4071 Australia

**Keywords:** Cell biology, Microbiology, Structural biology, Gastroenterology

## Abstract

Epithelial damage and loss of barrier integrity occur following intestinal infections in humans and animals. Gut health was evaluated by electron microscopy in an avian model that exposed birds to subclinical necrotic enteritis (NE) and fed them a diet supplemented with the probiotic *Bacillus amyloliquefaciens* strain H57 (H57). Scanning electron microscopy of ileal mucosa revealed significant villus damage, including focal erosions of epithelial cells and villous atrophy, while transmission electron microscopy demonstrated severe enterocyte damage and loss of cellular integrity in NE-exposed birds. In particular, mitochondria were morphologically altered, appearing irregular in shape or swollen, and containing electron-lucent regions of matrix and damaged cristae. Apical junctional complexes between adjacent enterocytes were significantly shorter, and the adherens junction was saccular, suggesting loss of epithelial integrity in NE birds. Segmented filamentous bacteria attached to villi, which play an important role in intestinal immunity, were more numerous in birds exposed to NE. The results suggest that mitochondrial damage may be an important initiator of NE pathogenesis, while H57 maintains epithelium and improves the integrity of intestinal mucosa. Potential actions of H57 are discussed that further define the mechanisms responsible for probiotic bacteria’s role in maintaining gut health.

## Introduction

**Figure 1:**
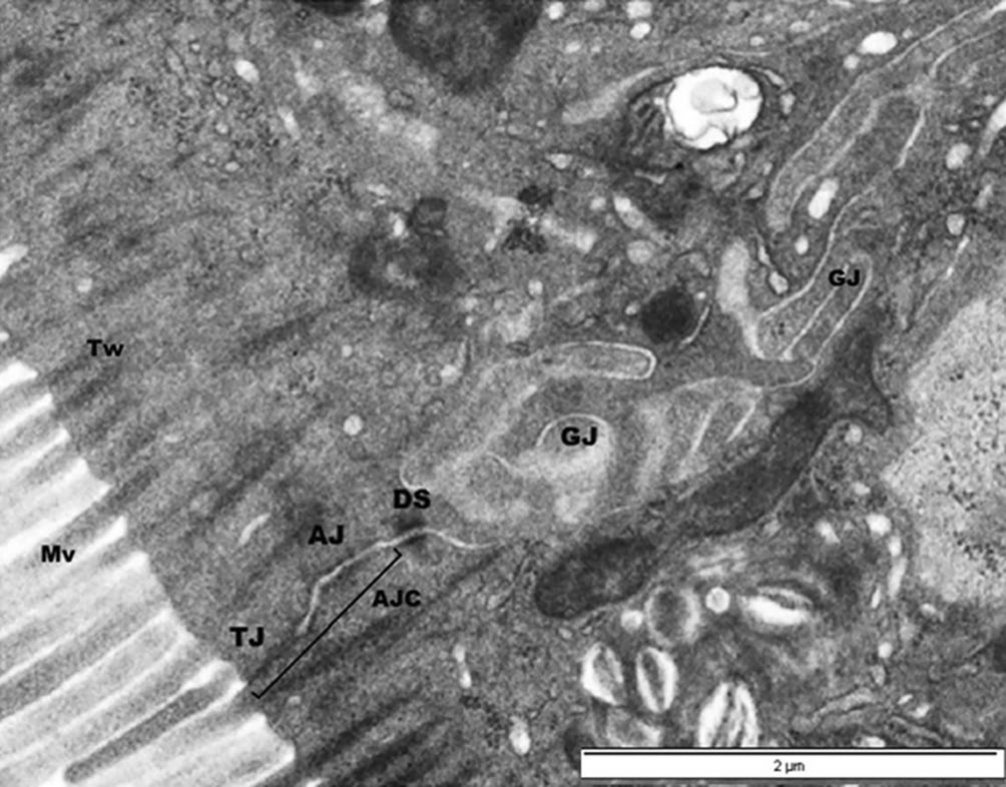
Transmission electron micrograph of a normal apical junctional complex (AJC), the structure between two adjacent enterocytes from the ileal epithelium region of a 21 d old chick. The AJC between neighboring cells is composed of TJ followed by AJ and DS. Identified are: microvilli (Mv), terminal web (Tw), tight junction (TJ), adherens junction (AJ), desmosome (DS), gap junctions (GJ). Scale bar, 2 µM.

As in mammals, the intestinal epithelium in birds is a single layer of cells, comprising columnar absorptive cells (enterocytes), goblet and enteroendocrine cells, and various immune cells. The intestinal mucosa projects into the gut lumen via villi, which are composed of an epithelial layer, a core of underlying lamina propria (containing the microvasculature) and a thin layer of smooth muscle (muscularis mucosae). Each villus serves as a functional absorptive unit^1^. The intestinal mucosa is covered by mucus, a complex hydrated gel that protects epithelial cells from chemical, enzymatic, microbial, and mechanical damage. The epithelium and its mucus layer permit the selective movement of ions, nutrients and water, but restrict the translocation of microbes and toxins from the lumen^2^. In particular, intercellular junctional complexes, which are specialized regions of contact between the plasma membranes of epithelial adjacent cells, are crucial for normal intestinal epithelium functioning^3^. Electron microscopy demonstrates that intercellular junctional complexes have four major components: the tight junction (TJ) or zonula occludens, the adherens junction (AJ) or zonula adherens, the desmosome (DS) or macula adherens, and gap junctions (GJ), or macula communicans (nexus). The TJ and AJ constitute the apical junctional complex (AJC)—the primary structure that regulates the intestinal barrier, and which is linked to the actin cytoskeleton. The DS and gap junctions are involved in cell–cell adhesion, and intracellular communication, respectively^4^. Figure 1 displays a normal junctional complex region from the chicken ileal epithelium. The AJC’s molecular composition, ultrastructure, and function can be evaluated with regard to physiological and pathological conditions, and its status can indicate paracellular barrier integrity loss^5^.

An intact epithelial barrier is essential for maintaining gut homeostasis in both humans and animals^6^. Ultrastructural modifications of intestinal mucosa occur in response to dietary changes^7^, including the presence of antigens in the diet, and changes in the composition of the gut bacterial community^8^. Ultrastructural examination of the epithelium, in particular enterocyte cells, and their constituents, such as mitochondria, lysosomes, endoplasmic reticulum, tight junctions and microvilli, can reveal the state of intestinal health. Other cells and molecules of the epithelial barrier unit include immune competent cells, i.e. intraepithelial lymphocytes (IEL) and tissue-related immune cells, and their products, cytokines and antimicrobial proteins. Moreover, mucus, and mucus-linked specific commensal bacteria, play important roles in the regulation of intestinal immunity and physiology^9^.

Disruption of the epithelium and disintegration of villi occur following enteric infections, and necrotic enteritis (NE) is an example of an infection disease that can cause severe damage to the small intestinal mucosa in chickens^10^. Birds with subclinical NE do not display clinical signs of disease, but can experience malabsorption and poor growth^11^. Partial or complete damage of microvilli and enterocyte necrosis has been reported during spontaneous NE caused by *Clostridium perfringens* (*Cp*)^12^. Enteric pathogens can also change the morphology and functionality of tight junctions^13–15^. In mice, intestinal infections cause disruptions of mitochondrial morphology and activity^16^. Similar changes are likely to occur during subclinical avian NE, however there have been limited reports of intestinal ultrastructure changes in NE disease^12,17^. Kaldhusdal et al.^12^ reported vesiculation and blebbing of the luminal cell membrane, and degenerative changes of cytoplasmic organelles, while Olkowski et al.^17^ observed disruption and fragmentation of intercellular junctional complexes, and increased cytoplasmic vacuolization, both features of ongoing necrosis.

Probiotic supplementation has shown promising results against various enteric pathogens in humans and animals, including prevention and treatment of necrotizing enterocolitis (NEC) in premature infants. In NEC, probiotics enhance the mucosal barrier by increasing the production of mucus, inhibiting bacterial translocation, and strengthening tight junctions^18–20^. The most commonly used model for bacterial intestinal infections in broiler chicks is the NE challenge disease model, and, for the current study, a subclinical NE model was employed. Our recent research demonstrated significant beneficial effects on intestinal mucosa maintenance and improvement of feed efficiency in a subclinical NE disease model in chickens supplemented with the probiotic *Bacillus amyloliquefaciens* strain H57 (H57)^21^. For the present study, electron microscopy was employed to evaluate intestinal villus ultrastructure and epithelium integrity of 21 day-old chickens exposed to NE and fed the probiotic H57. The aim was to gain greater insight into the pathogenesis of NE, and the mode of action of the H57 on maintaining intestinal mucosa integrity and function.

## Results

**Figure 2:**
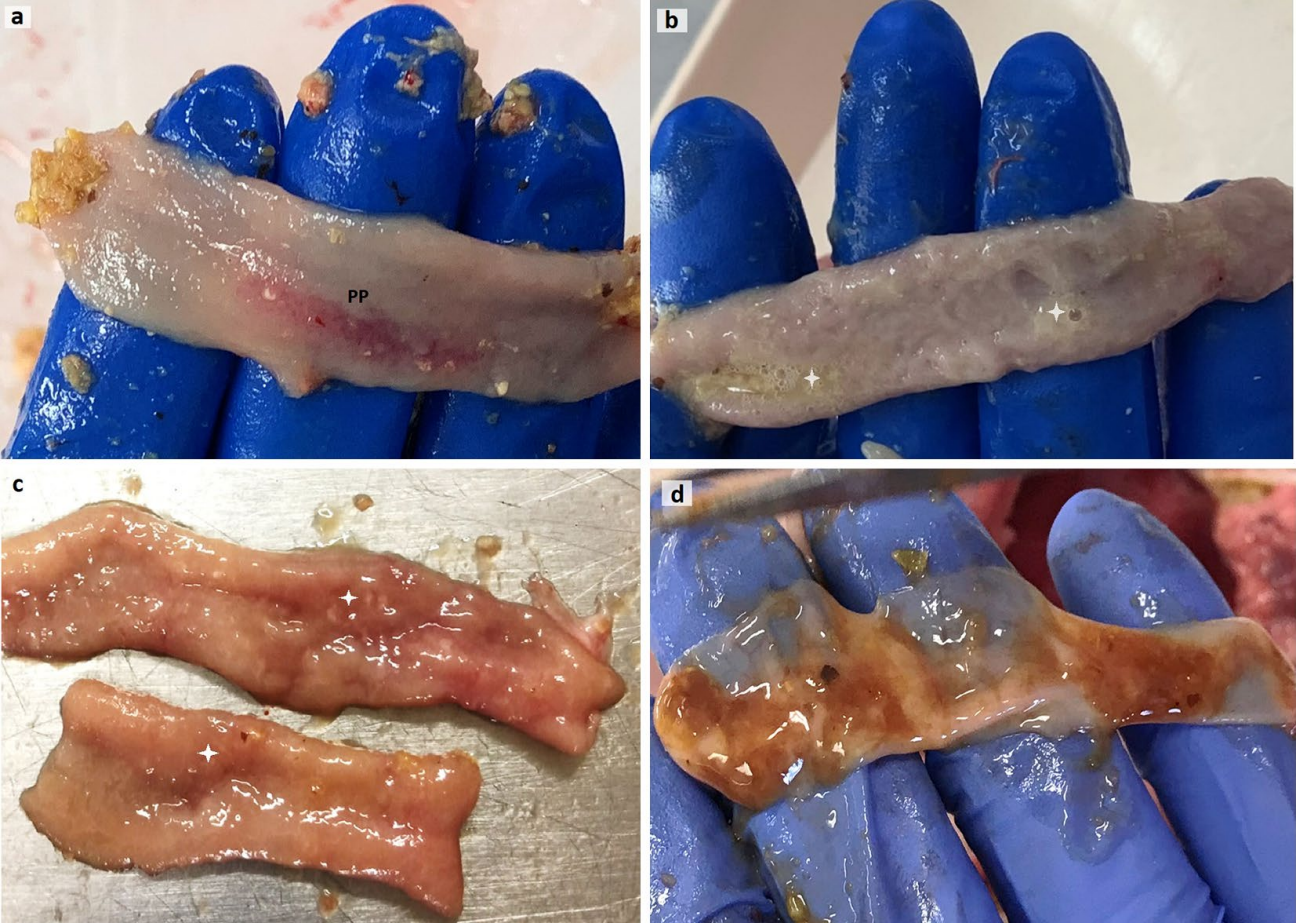
Gross pathology of the ileum from 21 d old chicks displaying mucosal changes characteristic for necrotic enteritis NE disease. Images of ileal mucosa from: a control chick showing normal mucosa and a hyperemic area representing Peyer’s patches (**a**); and from NE-challenged chickens: a watery content with moderate amount of gas (**b**), a creamy content covering hyperemic mucosa and some focal necrosis (**c**), thinning of intestinal wall, numerous necrotic foci and a bile-mixed content (**d**). PP is Peyer’s patches, white stars in (**b**) identify gas presence, and in (**c**) necrotic foci.

All chicks appeared clinically normal. In necropsy, chicks from control and H57 treatments demonstrated a greyish-pink, smooth, shiny and healthy mucosa, depicting some thickening bright red areas identified as mucosal lymphoid tissue inductive sites or Peyer’s patches (Fig. 2a). However, chicks co-infected with *Eimeria* and *Cp* (or NE-challenged birds) displayed mild focal intestinal lesions typical of NE on post-mortem examination, confirming subclinical NE disease (Fig. 2b–d). A few co-infected birds fed H57 (from NE & H57 group) displayed very mild signs of infection, such as hyperemic patches or scattered petechial areas in the mucosa, which are more likely to be signs of slight local transitory inflammation.

### Villus alterations

**Figure 3:**
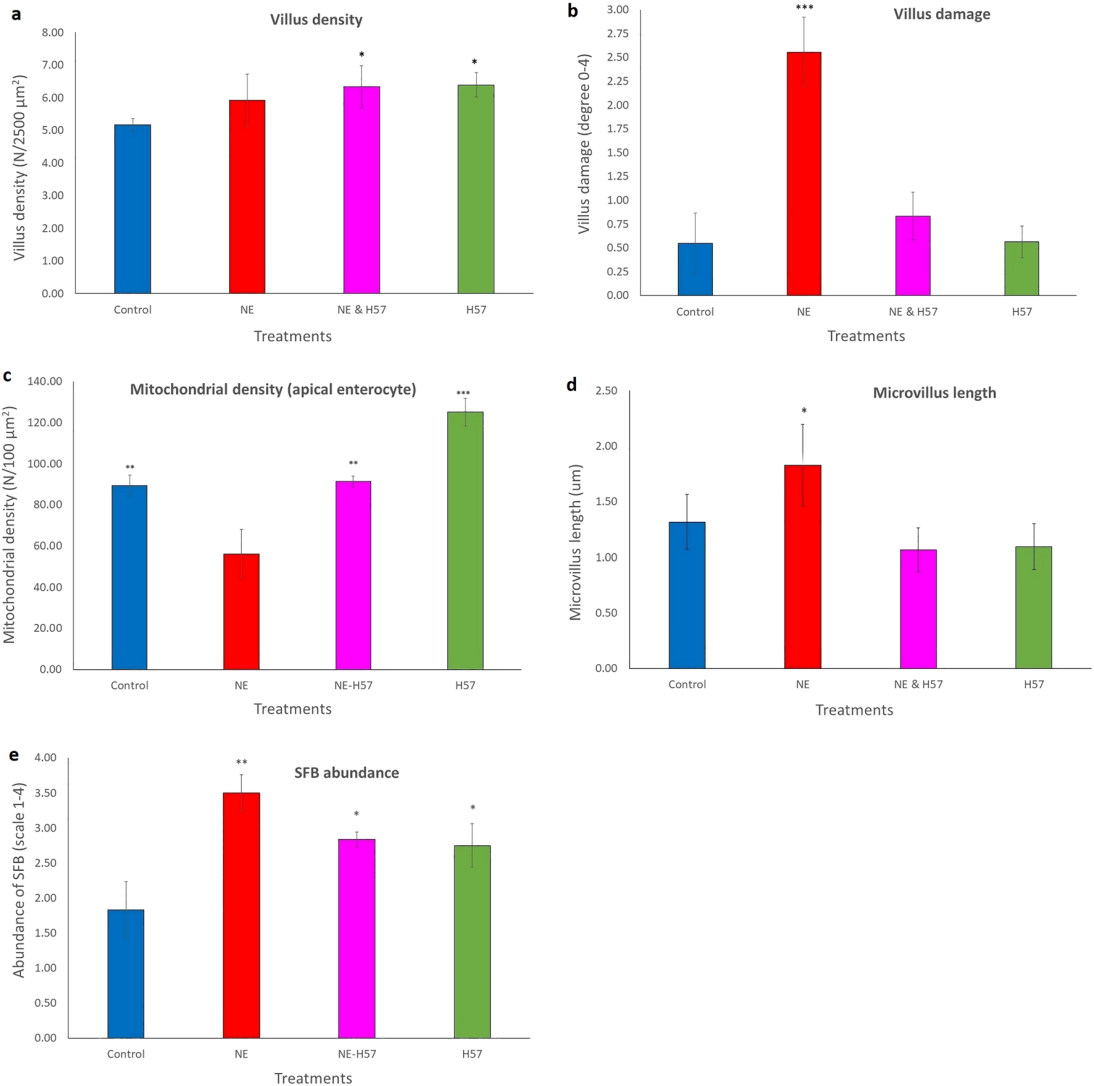
The effect of probiotic H57 on the villus density (**a**) and damage (**b**), SFB abundance (**c**), mitochondrial enterocyte density (**d**), and microvillus length (**e**) from 21 d old chicks exposed to necrotic enteritis. Control chicks were fed a basal diet (not supplemented with the probiotic H57) and not treated with any pathogen; NE chicks received a co-infection with *Eimeria* vaccine and *Clostridium perfringens* (*Cp*) and were fed a basal diet not supplemented with the probiotic H57; NE & H57 chicks were exposed to *Eimeria* vaccine and *Cp*, and fed a basal diet supplemented with the probiotic H57; H57 chicks were not treated with any pathogen, but fed the control diet supplemented with the probiotic. Error bars indicate SD, while asterisk indicates statistical significance (*p < 0.05; **p < 0.01; *** *p* < 0.001).

**Figure 4:**
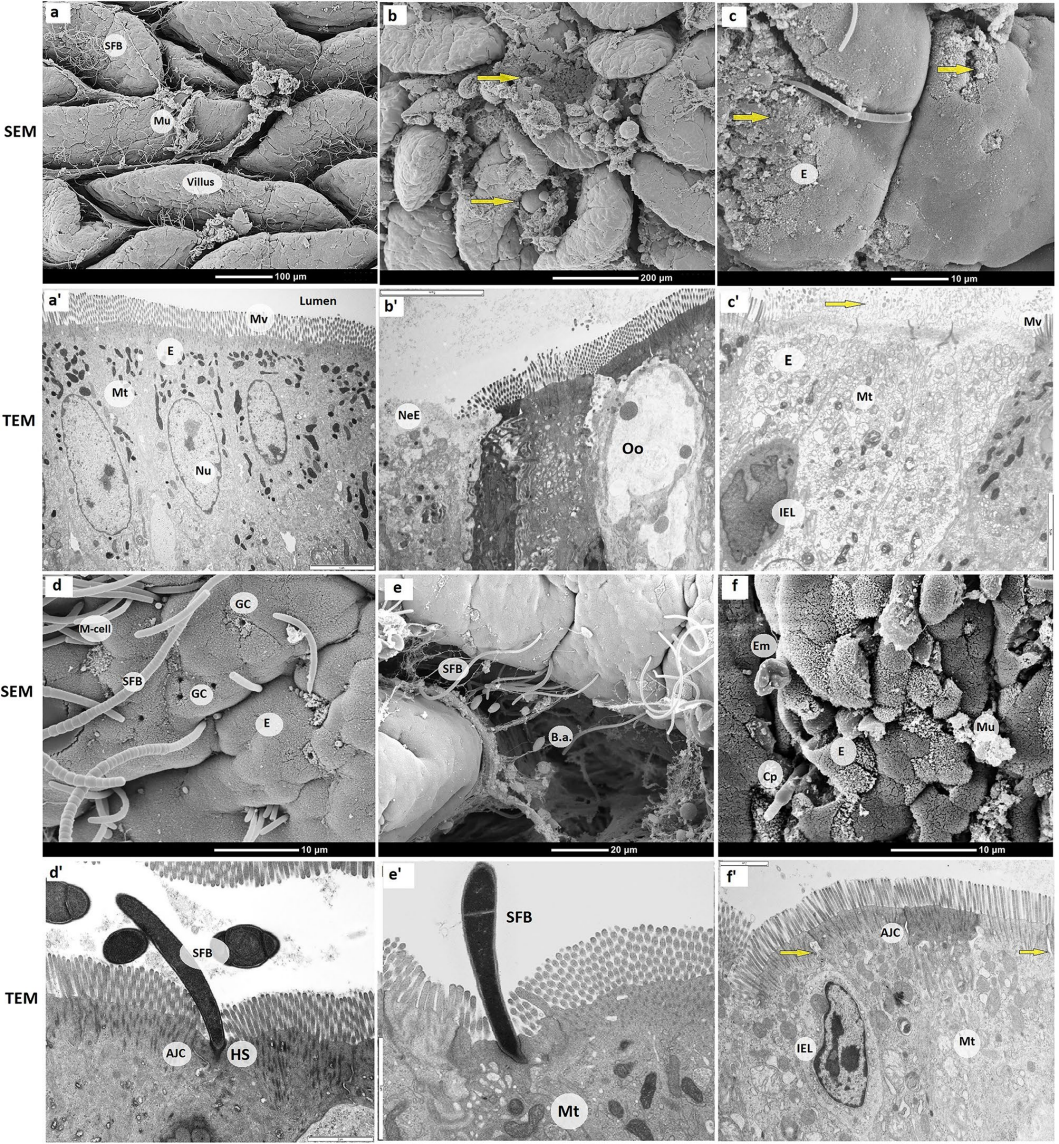
Scanning and transmission electron micrographs of the villi, ileal epithelial cells and other ultrastructural features from 21 d old chicks. Images showing ileal epithelium of a H57 (**a** & **a**’), NE (**b** & **b**’, **c** & **c**’, and **f** & **f**’) and NE & H57 (**d** & **d**’ and **e** & **e**’) birds. SEM and TEM images of each treatment are taken from the same sample (e.g. **a** & **a**’). Note, images of control and H57 birds were very similar in terms of villus or cellular damage, therefore only an image from H57 bird is shown in this figure. Figures 5a and 6a show images from a control bird. H57 chicks were fed a basal wheat-soybean diet supplemented with Bacillus amyloliquefaciens strain H57 at an average dose of 2.48 × 108 CFU/g feed; NE chicks were exposed to a coinfection with a high dose of Eimeria spp. vaccine and Clostridium perfringens (Cp), while NE & H57 chicks were exposed to NE and fed the control diet supplemented with the probiotic. Labelled are on images: **a** &** a**’, the villus, mucus (Mu), segmented filamentous bacteria (SFB), enterocyte, mitochondria (Mt), nucleus (Nu) and microvilli (Mv); images **b** & **b**’ and **c** & **c**’ from NE birds, yellow arrows show damage of villus tips and damage of epithelium and microvilli; enterocyte undergoing necrosis (NeE), Eimeria oocyte (Oo) and damaged mitochondria (Mt) and microvilli (Mv). In images **d** & **d**’ and **e** & **e**’ identified are a microfold cell (M-cell), goblet cells (GC), segmented filamentous bacteria (SFB) with the holdfast segment (HS), and Bacillus amyloliquefaciens (B.a.), while in images **f** &** f**’ displayed are Cp and an Eimeria sporulated oocyte (Em), mucus (Mu) and enterocyte (E), apical junctional complex AJC (yellow arrow), swelling and vacuolization of mitochondria (Mt), and intraepithelial lymphocytes (IEL). For TEM images scale bars are: **a**’, **b**’, and **c**’ = 5 µM; **d**’, **e**’ and **f**’ = 2 µM.

Data on villus density and damage collected from SEM images are presented in Figs. 3a,b, respectively. Ileal villus density was greater in H57 and NE & H57 birds compared with control birds (6.4, 6.3 vs 5.2 villi/2500 µM^2^; *p* < 0.05). Figure 4 presents SEM and TEM images of ileal tissue from the same treatment and sample ID; images from H57 birds (4a,a’), NE birds (4b,b’,c,c’), and NE & H57 birds (4d,d’,e,e’). Both SEM and TEM examinations demonstrate villus damage in NE birds, and no damage or restricted superficial damage in control, H57 and NE & H57 birds. As shown in Fig. 4a, normal ileal mucosa in a H57 fed chick forms tongue-shaped villi, which in many cases are covered by a layer of mucus and segmented filamentous bacteria (SFB). In NE chicks, mild to moderate pathological changes, as well as areas showing necrosis (Figs. 4b,b’,c,c’) demonstrate damage to the mucosa and support gross anatomy observations (i.e. diagnosis of subclinical NE). SEM of the ileal mucosa revealed focal erosions of epithelial cells, exposure of lamina propria (Fig. 4b,b’), and villous atrophy (Fig. 4b). The degree of damage was significantly greater (*p* < 0.001) in NE birds (graded as 2.6 degrees) when compared with all other treatments (Fig. 3b). NE & H57 treated birds did not display significant alterations of the villi (damage was graded 0.8 degrees).

### Enterocyte morphological alteration

**Figure 5:**
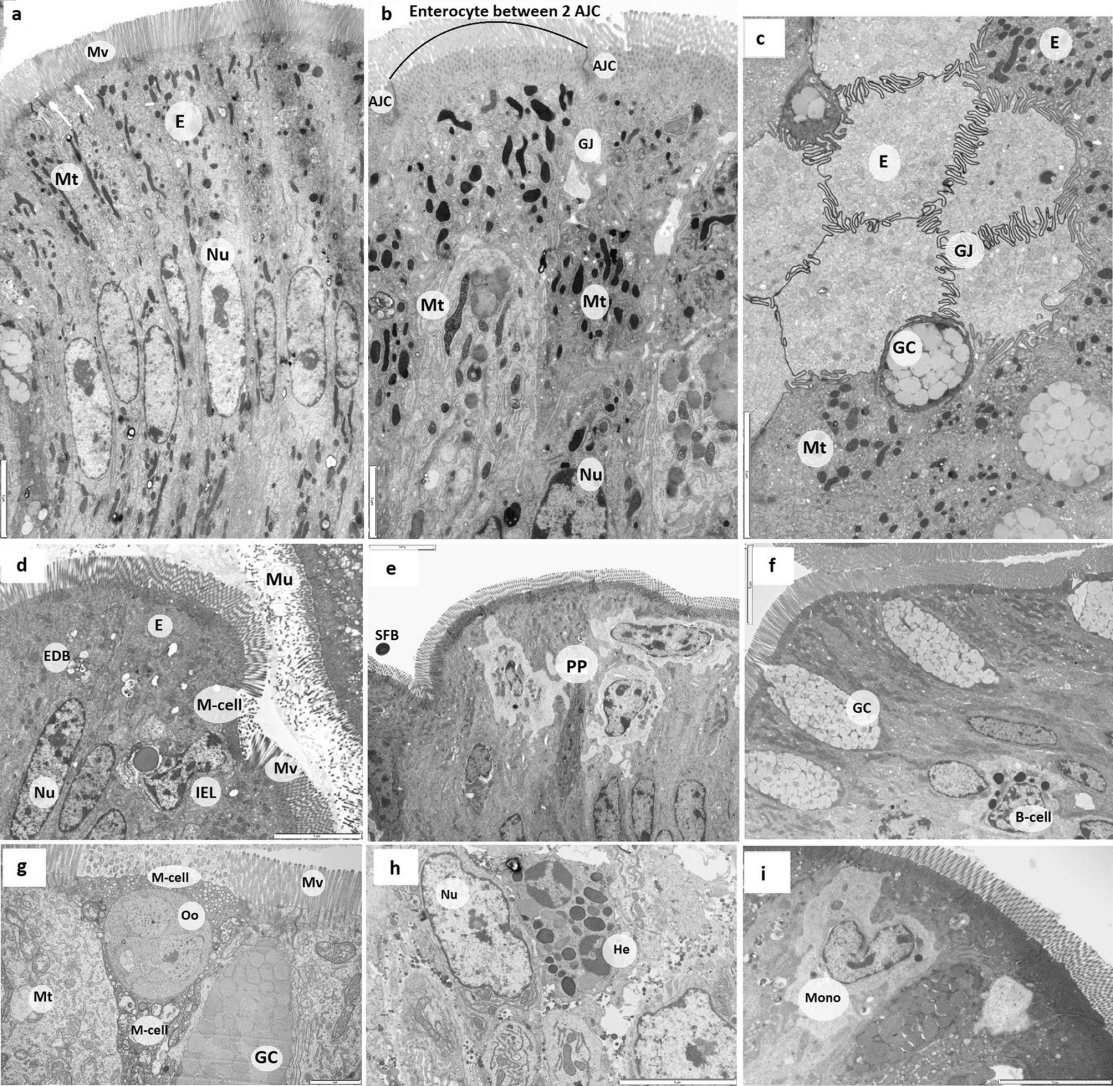
Transmission electron micrographs showing the architecture of intestinal epithelial cells and several types of immune cells in the ileum from 21 d old chicks. Image **a** is from a control chick, **b** and **f** are from a NE & H57 chick, and **c**, **d**, **e**, **g**, **h** and **i** from NE chicks. Images (**a**, **b** & **c**) show the ultrastructure of ileal epithelial cells, enterocytes (E), and their content: nucleus (Nu), mitochondria (Mt), microvilli (Mv) and the apical junctional complex (AJC) and gap junction (GJ). Image a show actin condensation in the AJC area (white arrows). Identified also are goblet cells (GC) that secrete mucin granules providing the mucosal surfaces with a thick mucus layer (Mu). Note a cross-sectional image of villus tip (image c) showing some light and dark enterocytes, and GC and GJ between adjacent cells. Images (**d**, **e**, **f**, **g**, **h** and **i**), show several leukocyte populations that can be found in the epithelium of the ileum, including intraepithelial lymphocytes (IEL) and microfold cells (M-cells). M-cells are located in the epithelium covering mucosa-associated lymphoid tissues, such as the Peyer's patches (PP, image **e**), and are specialized to sample macromolecules (antigens and pathogens) and transport them to macrophages and other immune cells to process. While the enterocytes possess a dense brush border with long, thin microvilli, M cell have apical microfolds. Image d shows the M-cell bordered by enterocytes and with an engulfed IEL; also the presence of electron dense bodies (EDB) demonstrating ingested bacteria, while image g shows the M-cell that has engulfed an *Eimeria* oocyte (Oo). Other leukocytes identified are B-cells (or plasma cell) which produce secretory immunoglobulin A (sIgA) a glycoprotein transported across intestinal epithelial cells into gut secretions (image **f**), and heterophils (He), which are recruited to inflammatory site to clear pathogens (image **h**); mononuclear cells (Mono), e.g. macrophage can also migrate to intraepithelial compartment (image **i**). Scale bars are: **a**, **c**, **d**, **e**, **f**, **g** and **i** = 5 µM; **b** = 2 µM and h = 3 µM.

Enterocytes and other epithelial cells, including goblet cells, IEL, heterophils, and M-cells were identified in all samples (Fig. 5a–g). Enterocytes’ sub-structures (microvilli), cytosolic organelles (mitochondria), and various cytoplasmic inclusions, including *Eimeria* oocytes (Fig. 5g), electron dense bodies and endolysosomes containing ingested bacteria (Fig. 5d), were also observed. In control, H57, and NE & H57 birds, most mitochondria were round or elongated, with little or no structural damage (Fig. 5a,b). In NE birds, mitochondria were irregular in form, containing electron-lucent regions of matrix, and indistinct, swollen or damaged cristae (Figs. 4c’ and  5g). Enterocyte mitochondria were more abundant in H57 treated birds (*p* < 0.001) compared with other groups, including controls (Fig. 3c). Birds in NE & H57 group also had greater mitochondrial density (75/100 µM^2^; *p* < 0.05) than NE-challenged birds (56/100 µM^2^), similar to control chicks.

There was a significant difference in microvilli length, with birds in the NE group having longer (*p* < 0.05) microvilli (approx. 1.6 µM) than all other treatments (approx. 1–1.2 µM) (Fig. 3d). Interestingly, microvilli in both H57 treatments appear shorter and thicker than microvilli of other treatments (data on thickness not collected). Microvilli were well-maintained in control birds and in H57 and NE & H57 groups. In NE birds, some microvilli damage was observed (Fig. 4b’,c’).

### Apical junctional complex (AJC) changes

Change in AJC length and morphology were investigated with TEM, and data are presented in Table 1. The length of AJC was shorter in NE birds compared with controls (0.88 µM vs. 1.07 µM; *p* < 0.05), and H57 and NE & H57 treatments (1.12 µM in H57, and 1.00 µM in NE & H57 birds). Dilatations within the AJC were evident in the AJ and DS regions (Fig. 4f’). TJ was visible but of normal morphology in most specimens, therefore only the frequency of dilatation (sacculation) of AJ and DS was measured. Exposure to NE increased dilatation of AJ, which were saccular in 47% of cases, while in NE & H57 birds only 20% of AJ were saccular. In control and H57 birds, AJ and DS were mainly of the linear form, in a few cases the DS was slightly dilated (Table 1). A greater frequency of actin condensation (*p* < 0.05) was found in NE and NE & H57 birds, compared with control and H57 birds.

**Table 1:** Apical junctional complex (AJC) length and morphology sourced from TEM micrographs of 21 d old chicks.

Group/treatment	AJC length (nM)	AJC qualitative description and frequency (%)^1^	Actin condensation degree & frequency (%) along AJ^2^
1. Control	1.07 ± 0.19^ab^	Linear (83.3) Slightly dilated in DS (16.7)	No (23.3) Minor (76.7)
2. NE	0.88 ± 0.15^d^	Linear (23.3) Slightly dilated in DS (30) Saccular (46.7)	Minor (23.3) Major (76.7)
3. NE & H57	1.00 ± 0.12^bc^	Linear (63.3) Slightly dilated in DS (16.7) Saccular (20)	Minor (33.3) Major (66.7)
4. H57	1.12 ± 0.13^a^	Linear (86.7) Slightly dilated in DS (13.3)	Minor (100)

Groups are identified as: control (chicks were not treated with any pathogen and fed a basal diet not supplemented with the probiotic H57); NE (necrotic enteritis) chicks received a co-infection with *Eimeria* vaccine and *Clostridium perfringens* (*Cp*) and were fed a basal diet not supplemented with the probiotic H57; NE & H57 chicks were exposed to *Eimeria* vaccine and *Cp*, and fed a basal diet supplemented with the probiotic H57; H57 chicks were not treated with any pathogen but fed the control diet supplemented with the probiotic.

^1^The number in brackets represents the frequency (%) of the qualitative description (linear vs. slightly dilated vs. saccular) found in the images observed.

^2^The number in brackets represents the frequency (%) of the actin condensation degree (no vs. minor vs. major) found from the images observed.

### Abundance of segmented filamentous bacteria (SFB)

**Figure 6:**
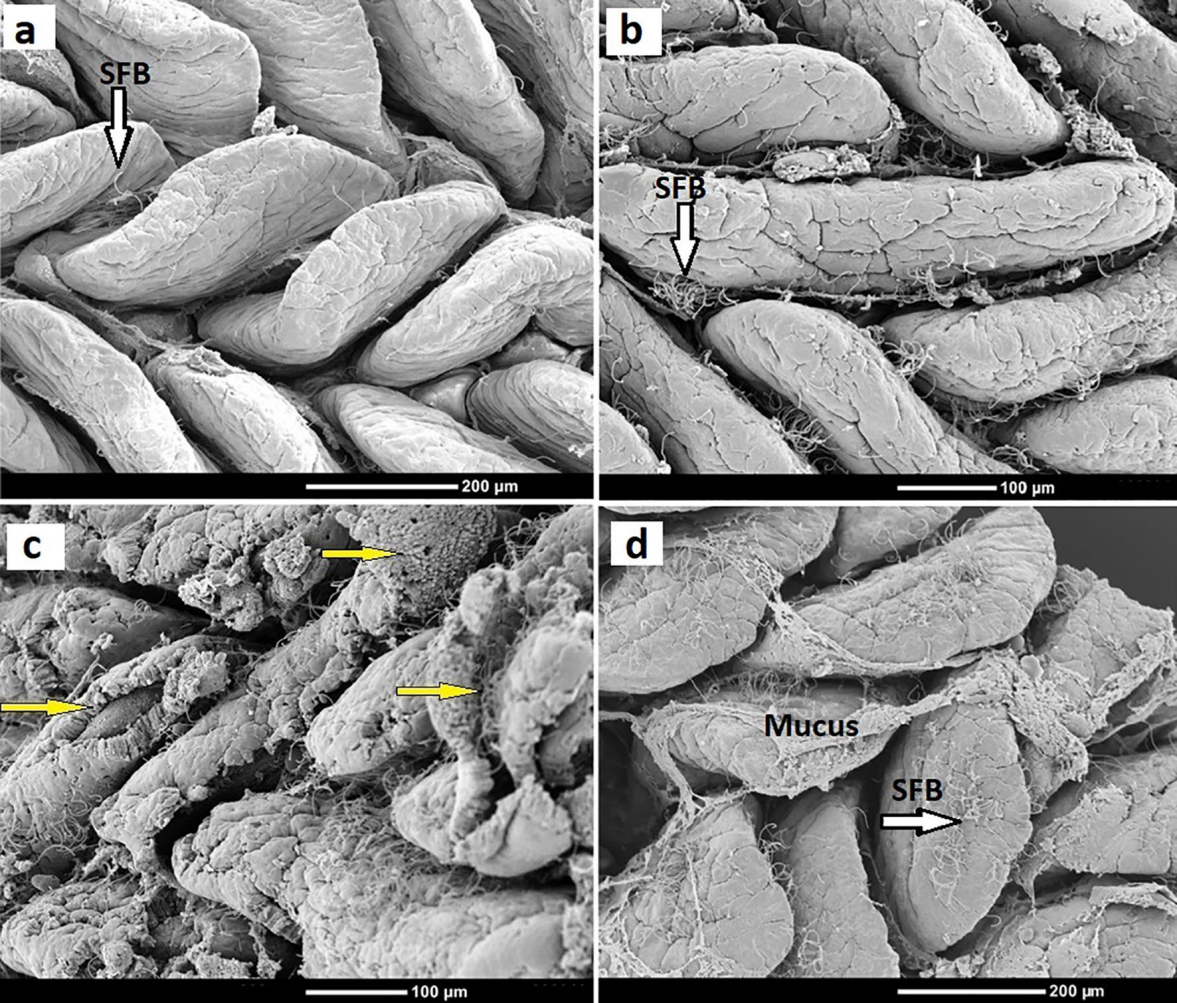
Scanning electron micrographs of ileal mucosa of 21 d old chicks demonstrating SFB, mucus and villus damage. Images looking from the top of mucosa, with examples showing segmented filamentous bacteria (SFB) colonization on the ileal villi (white arrows): low abundance of SFB (**a**) from a control chick, moderate abundance of SFB (**b**) from a H57 chick, and high abundance of SFB (**c**, **d**) from a NE-challenged and NE-H57 chick, respectively. Shown with yellow arrows are villi damage (**c**) in a NE-challenged chick, and mucus layer (**d**).

The abundance of SFB attached to villi was also assessed from SEM images and was greater (*p* < 0.05) in NE-challenged, NE & H57, and H57 birds in comparison with control birds (Fig. 3e). All birds (except control birds) showed moderate to high numbers of SFB over the ileal mucosa (Fig. 6b–d). However, in both probiotic treated birds, the average number of SFB was moderate and significantly lower (*p* < 0.05), than in NE-challenged birds (Fig. 3e). TEM and SEM demonstrated attachment of SFB to the apical membranes of epithelial cells (Figs. 4d,d’ and e). In some cases, SFB extended from an enterocyte into intimate association with a M-cell (Fig. 4d) or were mixed with mucus (Fig. 6d), and bacterial clumps or debris (Fig. 6c). SFB membrane (holdfast segment) was observed extending close to mitochondria or in proximity to an AJC area (Fig. 4d’,e’).

## Discussion

Ultrastructural evaluation of gut mucosa facilitates an insight into mechanisms specific to host–pathogen, host-probiotic, and pathogen-probiotic interactions. The intestinal mucosa is a complex frontier-barrier structure, with the ability for rapid regeneration that is essential for maintaining barrier integrity^19,22^. The changes we have observed occurring in the mucosal ultrastructure identify potential key regulatory structures and cells associated with damaged or healthy intestinal mucosa. In this study, a subclinical avian NE disease model was experimentally induced, and the ultrastructure of ileal mucosa evaluated in chicks supplemented with the probiotic H57. In a previous investigation, it was shown that birds exposed to NE and fed the diet with H57 had a significant improvement in feed utilization compared with the NE birds^21^. This effect could have been due, in part, to maintenance of gut barrier integrity.

**Figure 7:**
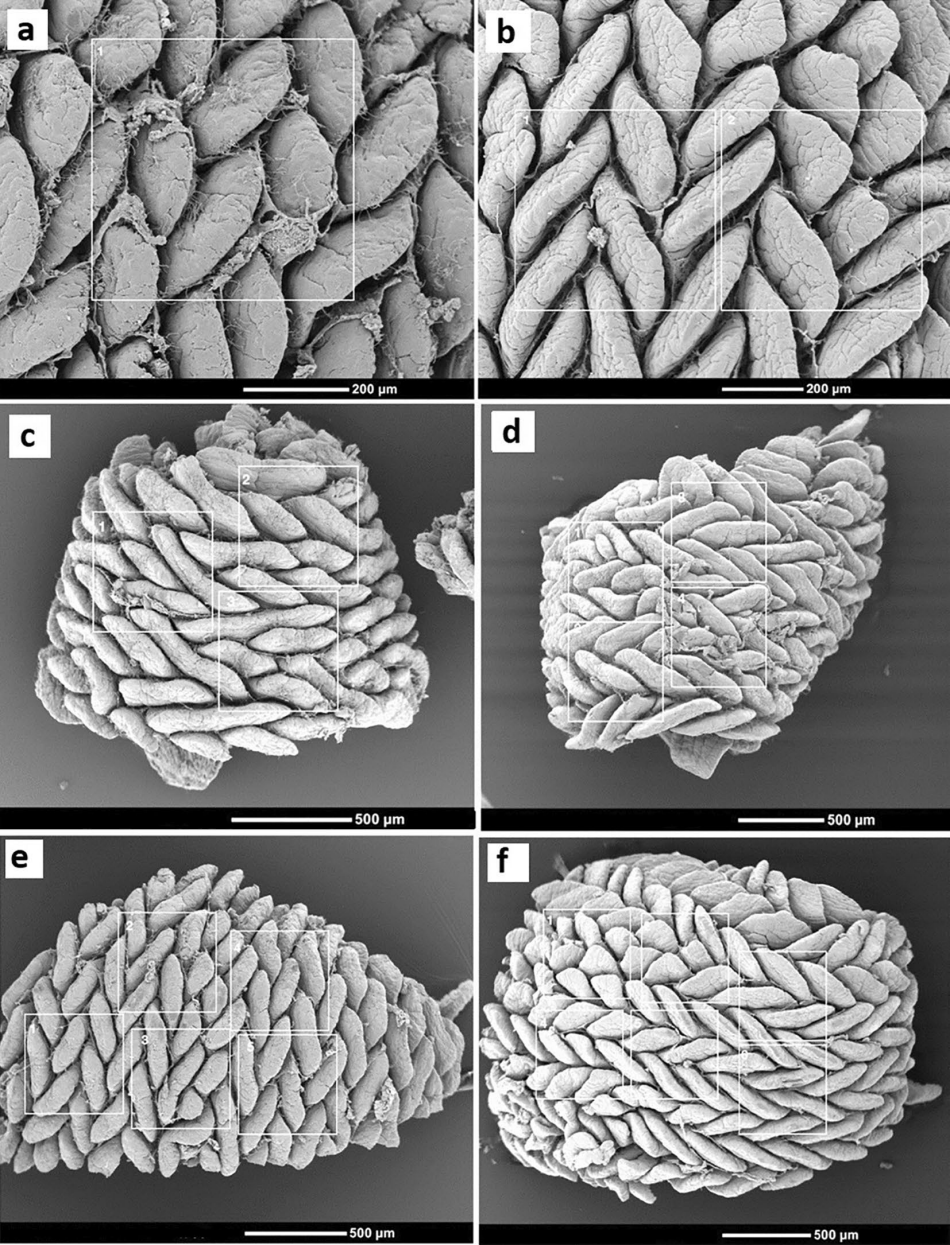
Scanning electron micrographs of villi from the ileum of 21 d old chicks. Shown are image examples that were employed to measure villus density. Images from different treatments fitting one (**a**) or more (**b**–**d**) squares. Villi within squares were counted and density was expressed as the number of villi/2500 µM^2^.

The findings of the current study provide new insights into the pathogenesis of subclinical NE and effects of probiotic H57 on intestinal health. When compared to NE birds that experienced epithelial damage and necrosis (Figs. 3b and 4b,b’,c,c’), the NE & H57 birds did not demonstrate any mucosal damage (Figs. 3b and 4d,d’,e). As shown in the SEM images (Fig. 6a, 7a–f), the ileum samples demonstrate a high density of tongue-shaped villi and a zigzag pattern, in most cases suggesting a wave shape. Interestingly, a zigzag pattern was more often seen in samples from H57 birds (Fig. 7b,f), however this was a general observation, and not quantified. This organization of the villi promotes nutrient absorption as it increases digesta retention time and allows more contact of nutrients with the absorptive surface of the intestinal epithelium^23,24^. Significant villi damage was observed in NE birds (Fig. 3b), whereas villi density was only slightly affected by treatment (Fig. 3a). Birds fed the probiotic had a small increase in villus density, compared with control birds. Increased villous density happens normally with age, however, it can be stimulated by rises in digestive enzyme activity. *B. amyloliquefaciens* (and H57) is known to produce extracellular enzymes^25–27^ for metabolic support, which could have initiated the slight increase in villi density.

SEM and TEM confirmed gross necropsy observations of mucosal damage in NE birds (Fig. 2b–d). There was damage of the ileal mucosa with epithelial erosion and some coagulative necrosis in these birds (Fig. 4b’,c’). The mechanism of injury (i.e. acute coagulative necrosis) in NE is caused by clostridial toxins^28^, and it can affect scattered single villi, or one or more villi clusters, or may affect all the villi in a section. *Eimeria* meronts are known to pass through the microvilli membrane to re-infect more enterocytes and alter villus dynamics^29^. However, in NE, enterocyte lysis by coccidia enables *Cp* access to binding sites for colonization, and also provides cell debris for bacterial nutrition^30^. Changes in apical enterocytes may be induced directly by the effect of toxins, and or indirectly due to villus ischemia and subsequent coagulative necrosis^28^. In the present study, damage to villus tips or the entire villus in chicks exposed to subclinical NE was observed. It should be noted that our samples were collected on day 21, therefore changes to epithelium and lamina propria were not detected and are not discussed here.

Our findings suggest that morphological and density changes in mitochondria may potentially lead to cellular necrosis and lesions in subclinical NE disease. Maintenance of the epithelial barrier is an energy-dependent process, thus in the case of swollen, irregular, vacuolated, or cristae damaged mitochondria (as in NE birds), loss of adenosine triphosphate (ATP) generation and release of oxygen radicals in enterocytes may have occurred. Subsequently, apoptotic necrosis of enterocytes and impaired energy metabolism of epithelial cells may induce a variety of insults, including spread of infection^31–33^. Mitochondria are dynamic organelles that undergo structural alterations to meet changing needs of epithelial cells and to maintain gut homeostasis^34^. Enterocyte mitochondria are involved in the regulation of numerous aspects of cellular activity, including the regulation of gut functions such as intestinal barrier integrity and mucosal immune responses^35–37^. Damage to this organelle will result in decreased energy production and eventually cell death^36^.

Previous investigators had suggested that the primary morphological changes in NE commence at the basement membrane of epithelium, (i.e. proteolytic damage within the lamina propria), and progress towards the center of the villus^17,38,39^. In contrast, we think that enterocyte mitochondrial damage during NE is a prerequisite for subsequent cell death and necrosis, and suggest that NE instead could be an intestinal disease associated with mitochondrial damage and energy deficiency. Mitochondrial damage has not been reported previously for subclinical NE, but it has been argued for bacterial enterotoxins^28,31,33^. Loss of epithelial barrier integrity, epithelial cell apoptosis, and bacterial invasion have been demonstrated following mitochondrial dysfunction^36^. Degenerative changes and mitochondrial dysfunction, including oxidative stress and impaired ATP production, are found in the intestines of patients with inflammatory bowel disease (IBD)^40,41^. The direct targeting of mitochondria has been reported for toxins belonging to the clostridial toxin family^42^. After having gained access to the cytosol, *Clostridium difficile* toxin B interacts with mitochondria influencing the ATP-sensitive mitochondrial K^+^ channels^43^, and subsequently causing mitochondrial swelling, vacuolization and damage.

Enterocytes are the functional unit, and the major cell type in the intestinal epithelium, and have important roles in nutrient transport, metabolism, and epithelial barrier integrity maintenance^44^. Enterocytes are more than just an absorbing cell, they respond to antigens in the gastrointestinal tract^45^, and have a pivotal role in the cross-talk between the IEL and luminal agents^46^. In response to injury, enterocytes undergo apoptosis and are continuously renewed by stem cells, however in the case of deregulated epithelial replacement, a small physical opening in the intestinal barrier allows bacterial translocation, and local inflammation^47^. In the current study, enterocyte alteration (enterocyte damage and necrosis) and vacuolization of cytoplasm and organelles were found in NE birds. Multivesicular bodies were also present, confirming cellular paranecrosis and subsequent necrosis in these birds (Fig. 4b’,c’ and f.’). In many cases these changes were associated with the presence of pathogens (*Eimeria* oocytes and bacterial inclusions) within enterocytes and intraepithelial immune cells found between enterocytes (Figs. 4b’,f and  5h). It remains to be investigated how shedding and replacement of enterocytes was deregulated and caused intestinal barrier damage in birds with NE. The lack of ATP could have been a potential factor, as ATP is central for cell renewal^48^. Studies in humans (IBD) suggest that both mitochondrial dysfunction and increased gut permeability affect the overall competence of the intestinal epithelial barrier^41,49^, but the stimuli that initiate either process is not known. However, in the current study, enterocytes of NE & H57 birds were intact and with normal-appearing cytoplasm and mitochondria (Fig. 5b). Mitochondrial density on apical enterocytes in both H57 fed groups (H57, NE & H57) was increased, in comparison with control and NE birds, respectively. Probiotic H57 produces various types of extracellular enzymes, which could have been the initiator of improved mitochondrial activity, cellular metabolism, and bioenergetics, explaining improved feed utilization in H57 treated birds^21^.

Enterocyte microvilli are densely packed together to form the apical brush border^50^. They have important roles in increasing the apical surface area and facilitating absorption, and protecting against luminal pathogens^51^. This study showed that microvilli were significantly longer in birds exposed to NE (i.e. *Eimeria* & *Cp*), (Fig. 3d), and, in some instances, damaged (Fig. 4c’). Lengthening of microvilli can occur to compensate for reduced absorption^51^. Significant elongation of microvilli and the formation of bacteria-filled cavities within the epithelial surface happen in other intestinal infections, resulting in villus disruption^52–55^. However, in contrast with our results, earlier research reported that, in NE, the mucosal epithelium architecture, including microvilli, remained largely unaffected^17,34^, most probably due to an earlier sampling time.

Recent studies have suggested a strong association between the junctional complex and intestinal health^5,56–59^. The paracellular barrier in healthy intestinal tissue is characterized by high expression levels of TJ proteins and a low paracellular permeability (or a tight epithelium), while mucosal inflammation is frequently associated with decreased expression of junctional proteins (or a leaky epithelium)^58,60,61^. Numerous bacteria have been implicated in altering TJ, in particular *Cp* species. They use a potent enterotoxin that binds to two members of claudins, making them unstable^13^. Recent human studies have indicated both downregulation and upregulation of TJ proteins, in particular claudins, in patients with IBD^62,63^. Nevertheless, the role of these proteins in modulating TJ and epithelial barrier function is not fully understood.

In this paper, we demonstrate ultrastructural changes of AJC in a subclinical NE avian model. No morphological changes in TJ were found, however the widening (sacculation) of the AJC (in the AJ and DS parts) was significant (Table 1). AJ and DS link membrane and cytoskeletal components at discrete contact regions, and function in concert to orchestrate tissue organization and functionality^4^. Our study suggests that dietary addition of H57 in NE-challenged birds maintains the normal morphology of AJC (AJC were significantly longer and less saccular in NE & H57 birds compared with NE birds), and subsequently reduces intestinal damage. In NE birds, the length of AJC was decreased, presumably due to a lack of structural components (proteins) or decreased recruitment of AJ or TJ proteins. Altered or reduced expression of such proteins could favor the influx of luminal antigens and consequently cause inflammatory damage^60^. Signaling molecules and extracellular stimuli such as cytokines and nutrients regulate the AJC^64^, while pathogens target structural and regulatory components of the AJC^65,66^. Actin, as a part of the cytoskeleton, connects AJC into an integrated network. Numerous bacterial toxins recognize the actin cytoskeleton as a target, as microbes utilize the host cell cytoskeleton for many activities, such as attachment, entry, movement within and between cells, vacuole formation, and avoidance of phagocytosis^67–69^. There are toxins (including clostridia toxins) that modify the actin cytoskeleton and alter the function of the junctional complex, thus causing cell lysis and absorption of toxins by the vascular system^28,70^. As mentioned above, we did not find morphological changes in TJ, and agree with Jou et al.^71^ who demonstrated increased paracellular permeability without disruption of TJ protein organization. Another study, however, showed that increased paracellular permeability was associated with significant redistribution of the TJ proteins^64,72^. In the current study, we identified differences in actin filaments condensation in the AJ zone by TEM. There is normally little actin condensation evident at the TJ and AJ area^73^ as actin networks are in a dynamic steady state^74^. Actin condensation within the host cell occurs most probably due to bacterial-induced host cell signaling directly beneath the groups of adherent bacteria^75,76^. Our preliminary data demonstrate that exposure to NE triggers actin condensation, while in NE & H57 birds, the accumulation of actin was reduced. *Clostridium* toxins prevent actin filament polymerization (elongation), resulting in complete depolymerization of cellular actin and potentially cell death^32,77^.

A fascinating aspect of this study was the appearance and evaluation of the abundance of segmented filamentous bacteria (SFB). There were more SFB attached to villi in birds exposed to NE, NE & H57 and H57 compared with control birds (Fig. 6). SFB are intestinal commensal microorganisms ranging from 0.7 to1.8 µM in diameter and up to 80 µM in length^78^ (Fig. 4d,d’) that have important roles in host immunology and physiology^79,80^, such as regulating postnatal development and maturation of immune responses in the gut. Little is known regarding the diversity of the SFB group, but they are host specific^81^. They have gained attention due to their capacity to induce and stimulate multiple types of intestinal lymphoid tissues (Peyer’s patches and IEL) for the generation of T helper 17 (Th17) cell responses^82,83^. SFB are found on ileal mucosa, and stimulate Th17, which are capable of producing IL-17 cytokines with a proinflammatory role in the mucosal (IgA) immune response generation^79,84^.

In this study, chicks feed H57, with and without NE disease, had an increased abundance of SFB, when compared to control birds, however the increase in abundance was significantly less than in NE birds. It appears that H57 downregulated SFB abundance in birds exposed to NE. Probiotics favoring SFB could therefore have an effect in regulating the immune response in the gut^85,86^. There is evidence that probiotics have anti-inflammatory effects, as they downregulate IL-17 production and other proinflammatory Th17-secreted cytokines^87,88^. The IL-17 is beneficial in controlling dysbiosis in the gut, but may be harmful if dysregulated, therefore in the case of overgrown SFB, accumulation of Th17 cells in the ileum could lead to the damaging inflammatory effects^89^ as seen in NE birds. Chickens exposed to stressful condition that predisposes them to NE can be offered diets supplemented with probiotics, as an approach for limiting SFB expansion and the Th17-associated proinflammatory response. Probiotics (including H57 from this study) tend to induce a regulatory response in the context of inflammatory and some autoimmune diseases^90^.

## Conclusions

Our data highlight the potential role of the probiotic H57 in improving epithelial cell maintenance and integrity of the ileal mucosa during subclinical infection with NE disease (co-infection with *Eimeria* and *Cp*). When birds were exposed to NE, there was mucosal damage, and enterocyte cytoplasmic alteration and necrosis. In particular, mitochondrial morphology and density were impaired. In contrast, birds challenged with NE and fed the probiotic displayed intact villi with normal enterocyte morphology and well-maintained mitochondria. Our findings identify new ultrastructural features involved in the pathogenesis of NE, and unravel some mechanisms of probiotic action, including effects on mitochondria morphology and regulation of SFB abundance on the ileal mucosa. In NE-challenged birds exposed to the probiotic, colonization of the intestinal mucosa with SFB appears to improve their response to subsequent bacterial infection. Since the integrity of epithelial cells is energy-dependent, mitochondrial function is undoubtedly crucial to the maintenance of intestinal physiology. Therefore the role of mitochondria in gut health, in particular, enterocyte mitochondrial bioenergetics during intestinal infections needs to be further explored.

## Materials and methods

### Ethics

The experimental studies and procedures involving meat chickens were approved by the Animal Ethics Committee of the University of Queensland (SAFS/192/18). All the experiments complied with ARRIVE guidelines^91^ and were carried out in accordance with the Australian Code for the Care and Use of Animals for Scientific Purposes^92^.

### Birds and bird husbandry

Day-old male broiler chicks (Ross 308) were obtained from a commercial breeder (Aviagen Australia Pty Ltd). Birds were vaccinated against Marek’s disease, infectious bronchitis, and Newcastle disease at the hatchery. From day one, chicks were kept in an isolated, temperature-controlled room, in which the brooding temperature and a lighting regimen were as recommended by the breeding company^93^. The room was located in the Poultry Research Facility on Gatton Campus (University of Queensland), and was thoroughly cleaned and disinfected prior to bird placement. One hundred and ninety two chicks were individually weighed, and randomly placed in 24 cages (8 birds/cage), at a stocking density of 13 birds/m^2^. Chicks were fed an all-phase wheat-soybean-based mash diet (2900 kcal/kg ME, 24% CP, 0.9% Ca & 0.45% available phosphorus) from day-old until the end of the experiment (day 21). The basal diet was not supplemented with any antimicrobial growth promoters or coccidiostatics, and did not contain any other recognized substances with antibacterial properties. Feed and water was supplied ad libitum*,* except on treatment days, which required birds to have restricted water and feed supply (days 9 and 14). Strict biosecurity management practices were followed to prevent cross contamination between control and treated birds.

### Experimental treatments

Table 2 presents details of experimental groups and treatments for chicks from day 0 to day 21. Four treatments were employed in this study, each comprising six replicates with eight chicks per replicate. Birds assigned to the non-challenged group were fed the basal diet (control, group 1). A NE-challenged group was fed the basal diet (NE, group 2), a NE-challenged group was fed the basal diet supplemented with H57 (NE & H57, group 3), and a non-challenged group was fed the basal diet supplemented with H57 (H57, group 4). The H57 experimental diet was produced by adding H57 to the basal diet at an average dose of 2.48 × 10^8^ CFU/g feed (confirmed by microbiological analysis) and was fed to groups 3 and 4 throughout the experiment. Shini et al.^21^ has detailed the NE challenge, to which groups 2 and 3 were exposed. Briefly, birds challenged with NE were exposed to a coinfection with a commercially available anti-coccidiosis vaccine containing 4 strains of *Eimeria spp.* (i.e. viable oocysts of *E. acervulina, E. maxima, E. necatrix* and *E. tenella*) at a concentration of 1.6 × 10^4^ oocysts/mL and freshly prepared broth culture material containing a *Cp* suspension (1.76 × 10^8^ CFU/mL). On day 9 post-hatch, chicks were exposed to *Eimeria* vaccine (20 × the manufacturer's recommended dose, or 8000 oocytes/bird suspended in 0.5 mL PBS/bird) via drinking water, and 5 days later to a virulent *Cp* strain EHE-NE18 (CSIRO, Geelong, Australia) mixed in feed at a ratio 1: 1.5 (vol/wt).

**Table 2:** Experimental groups and treatment details for chicks from day 0 to day 21.

Groups and treatments	Days 0–8	Day 9	Day 10–15	Day 16–20	Day 21
1. Control (basal diet)	All groups fed their diets. Feed intake & body weight recorded on day 7 All groups tested for *Eimeria spp.* oocytes in the feces on day 8	PBS^2^	All groups fed their diets. Feed intake & body weight recorded on day 14 Sterile broth^3^	All groups fed their diets On day 20, all groups tested for *Eimeria spp.* oocytes in the feces	Feed intake & body weight recorded One bird/replicate (6/treatment) was euthanized for necropsy & tissue sampling
2. NE^1^ (basal diet)		*Eimeria* vaccine	Broth inoculated with *Cp*^3^		
3. NE and H57 (basal diet + H57)		*Eimeria* vaccine	Broth inoculated with *Cp*		
4. H57 (basal diet + H57)		PBS	Sterile broth		

^1^NE-exposed chicks received a coinfection with *Eimeria* vaccine on day 9, and *Clostridium perfringens* (*Cp*) on days 14 and 15.

^2^PBS and *Eimeria* vaccine was delivered in the drinking water (water was withheld for 3 hr prior treatments).

^3^Sterile broth and broth inoculated with *Cp* was mixed with feed and given to birds; feed was withheld for 5 hr prior treatments.

### Sampling and sample processing for electron microscopy

Immediately, after euthanasia, a 2-cm length of the ileal tissue (middle part), was rinsed with cold Dubleccos Phosphate Buffered Saline (DPBS) and then cut into very thin strips (1 mm × 2 mm). The tissue was fixed in 3% glutaraldehyde in 0.1 M phosphate buffer 7.4 at 4ºC. Subsequent processing was conducted using a microwave processor. For scanning electron microscopy (SEM), specimens were post-fixed in osmium tetroxide, dehydrated in ethanol, and critical point dried using an Autosamdri-815 series point dryer (Tousimis, Rockville), sputter coated with gold (using a SPI module sputter coater) prior to viewing with a Neoscope JCM-5000 SEM (JEOL, Japan). For TEM, tissue was post-fixed in 1% osmium tetroxide, dehydrated, and embedded in Epon resin. Ultrathin sections were stained with uranyl acetate and lead citrate and examined in a JEM1011 (JEOL Pty Ltd, Tokyo, Japan) microscope operating at 80 kV.

### Ultrastructural analysis and measurements

Using ImageJ software program (1.51 V)^94^, two observers took all SEM and TEM measurements independently. SEM images were employed to observe and measure changes on density and conditions of the villi. The density was determined utilizing squares (500 × 500 µM) overlaid on sets of photomicrographs. Adjustments for the scale were made, and Fig. 7A–F shows sample images from different treatments fitting one or more squares. Villi within squares were counted and density was expressed as the number of villi/2500 µM^2^. If villi were touching the four perimeter sides of a box, either the two outer sides with villi or two inner sides were counted. The same images were used to evaluate apical damage of villi. The degree of villus damage was graded using a scale developed by Gomide Junior et al.^95^, and modified for our needs (Table 3). To assess segmented filamentous bacteria (SFB) abundance, micrographs fitting only one square were used (Fig. 7a). The abundance of SFB attached to the ileal epithelium was assessed by counting the incidence of SFB colonized to one entire villus inside the box. The evaluation criteria was based on previous reports^78,96^, and graded as: absent or none (−); low density ( +), when less than five SFB attached to a villus were counted; moderate density (+ +), when between six and fifty SFB were found; and high density (+ + +), when > 50 SFB were counted (Fig. 6a–d).

**Table 3:** Criteria for the assessment of ileal villus damage from SEM micrographs, modified from Gomide Junior et al.^94^.

Criteria	Degree of damage
No apparent loss of epithelium (normal villi)	0
Small areas of epithelium loss on 1 to 3 villi	1
Small areas of epithelium loss on more than 3 villi	2
Large areas of epithelium loss exposing lamina propria, and presence of debris on 1 to 3 villi	3
Severe damage of the tip of villus on more than 3 villi, associated with shorten/thicken villi or villi atrophy	4

TEM images were used to assess the morphology of epithelial cells, in particular enterocytes and their content and features, such as cytoplasmic organelles (mainly mitochondria), microvilli and the AJC. The morphology and abundance of enterocyte mitochondria was assessed from photomicrographs of six enterocytes/sample or 36/treatment. Enterocytes with regular (uninterrupted) microvilli were evaluated in all occasions (Fig. 5a). Six individual microvilli/cell were measured from the tip of the microvillus to its attachment to the enterocyte membrane (3 cells/sample, or a total of 104 microvilli/treatment were evaluated). In each enterocyte, the area of the apical enterocyte (over the cell nucleus) was measured, and the number of mitochondria in this area was counted (Fig. 5a). The density of enterocyte mitochondria was calculated as the number of mitochondria/100 µM^2^ of cell area.

To evaluate changes in enterocyte AJC morphology, the length of AJC between two adjacent enterocytes, and their degree of separation (normal or linear vs. saccular) was determined. As suggested by Karcher and Applegate^22^ to maintain consistency in measurements, length of AJC was taken from the apical membrane, where the TJ began and included not only TJ and AJ, but also DS (Fig. 1). Only cells on the top portion of the villus tip with two adjacent AJC were used in the analysis (Fig. 5b). Therefore, measurements/image ranged from 2 to 6, and the total number of measurements/treatment was 30. Actin condensation degree and frequency (%) along AJ (Fig. 5a) was also evaluated from these images. Morphological observations were made for other epithelial cells, i.e. goblet cells (Fig. 5c,f), M-cells (Fig. 5d), heterophils (Fig. 5h) and intraepithelial lymphocytes (Fig. 5i).

### Statistical analysis

Data were analyzed using MiniTab 17 software. Comparisons between treatments were made with the one-way ANOVA procedure and Tukey’s multiple comparison test. Differences with *p* < 0.05 were considered significant. Level of significance is presented in relevant tables and figures. For statistical analyses, the SFB abundance was recorded as categorical data, thereafter transformed into numbers using an encoding technique in Minitab.

## Data Availability

All data generated and analyzed during this study are included in this manuscript and in the supplementary information files. Further details are available from the corresponding author upon request.
